# Practice of community pharmacists regarding dietary supplements in osteoarthritis management: a mystery shopper study in Iran

**DOI:** 10.1186/s40780-025-00492-9

**Published:** 2025-10-10

**Authors:** Fatemeh Dehghan, Fatemeh Dabaghzadeh

**Affiliations:** 1 Herbal and Traditional Medicines Research Center, Kerman University of Medical Sciences, Kerman, Iran; 2 Department of Clinical Pharmacy, Faculty of Pharmacy, Kerman University of Medical Sciences, Haft-Bagh Highway, P O Box: 76175-493, Kerman, 76169-13555 Iran

**Keywords:** Dietary supplements, Mystery shopper, Osteoarthritis, Pharmacist

## Abstract

**Background:**

Many osteoarthritis patients incorporate dietary supplements and nutraceuticals into their self-management strategies. Community pharmacists play a pivotal role in patient education by identifying potential drug interactions and guiding the evidence-based and safe use of dietary supplements. This study aims to assess the real-world practice of Iranian pharmacists in managing osteoarthritis, with a specific focus on dietary supplement counseling, using the mystery shopper method.

**Methods:**

This cross-sectional study was carried out in 225 active community pharmacies. Two female mystery shoppers performed a two-step scenario. In the first step, the mystery shopper requested an avocado/soybean supplement for knee pain from the pharmacist. In the second step, if the pharmacist did not inquire about the consumer or their medication history, the mystery shopper stated that the supplement was intended for her 64-year-old father, who was taking warfarin. All conversations were audio-recorded, and the pharmacists’ practice was documented.

**Results:**

Only 15 pharmacists inquired about the intended user. During step 1, only seven pharmacists successfully identified the potential warfarin– avocado/soybean interaction. In step 2, after receiving additional patient-specific details, the recognition rate increased to 29 pharmacists. However, no pharmacists provided further information about additional interactions, adverse effects, or contraindications related to avocado/soybean supplements. Only the seven pharmacists explained how to use the avocado/soybean supplement, while nine pharmacists obtained medical history, and eight pharmacists gathered medication history.

**Conclusion:**

Pharmacists provide limited and insufficient counseling on dietary supplements for osteoarthritis, highlighting a significant gap in patient education and the safe use of dietary supplements.

## Introduction

Osteoarthritis (OA) is a chronic, progressive joint disorder and is among the most prevalent musculoskeletal conditions worldwide. It occurs due to an imbalance between catabolic and anabolic processes, leading to cartilage degradation, joint pain, and diminished physical function [1]. Currently, OA affects over 500 million people globally and is a leading cause of disability, particularly among individuals aged 60 years or older [1, 2]. With increasing life expectancy, projections indicate that nearly 30% of individuals over 45 may be diagnosed with OA within the next decade [3]. The economic and social consequences of OA are significant, encompassing increased healthcare expenditures, loss of productivity, absenteeism, and early retirement [4]. In low- and middle-income countries, challenges such as limited access to early diagnosis and comprehensive care, compounded by low public awareness and underdiagnosis, further exacerbate the disease burden [5].

OA management primarily focuses on symptom relief and slowing of disease progression [3, 6]. While non-steroidal anti-inflammatory drugs (NSAIDs) remain the cornerstone of pharmacologic treatment, long-term safety concerns have spurred interest in alternative therapies, including dietary supplements. Studies indicate that 30%–69% of OA patients incorporate dietary supplements and nutraceuticals into their self-management strategies [7, 8]. According to the United States Food and Drug Administration (FDA), dietary supplements are formulations containing one or more nutritional ingredients such as vitamins, minerals, amino acids, or plant extracts, and are widely available without a prescription. Despite their popular perception as being natural and safe, issues regarding variable effectiveness, potential adverse effects, and possible interactions—especially among the elderly who often use a combination of prescription and nonprescription medications —remain significant [9, 10].

Community pharmacists play a pivotal role in patient education by identifying potential drug interactions and guiding the evidence-based and safe use of dietary supplements, particularly in countries like Iran, where supplement use is widespread [3, 11]. However, previous studies indicate that Iranian pharmacists’ knowledge and performance in advising on over-the-counter (OTC) medications and dietary supplements remain suboptimal [12, 13]. Moreover, data on pharmacists’ readiness to address the complex needs of OA patients in Iran are scarce. The mystery shopper (MS) methodology—wherein trained individuals simulate real-life clinical scenarios—has emerged as an effective tool for objectively assessing pharmacist practice, particularly in high-risk populations [4]. To the best of our knowledge, no previous study in Iran has applied this approach to evaluate community pharmacists’ performance in OA management. Therefore, this study aims to assess the real-world practice of Iranian pharmacists in managing osteoarthritis, with a specific focus on dietary supplement counseling, using the MS method.

## Method

### Ethics approval

This study was approved by the ethics committee of Kerman University of Medical Sciences, Kerman, Iran (Ethical approval code: IR.KMU.REC.1402.439). One month before data collection, pharmacy managers of the pharmacies were contacted by telephone to explain the study protocol and obtain verbal informed consent. Additionally, the pharmacists in charge signed a written informed consent form. They were informed that all interactions would be audio-recorded and that non-identifiable demographic information—such as age, gender, years of experience as a community pharmacist, and pharmacy owner—would be collected. During each visit, the MS verified the identity of the pharmacist responsible for patient counseling.

### Setting

This cross-sectional study was carried out in community pharmacies across Kerman, Iran, from October to November 2024. During this period, the city had a total of 225 community pharmacies distributed across various areas. Data collection was carried out during the evening hours, ensuring that one pharmacist was actively present at each pharmacy.

### Mystery shoppers and scenario

Two female pharmacy students, both aged 23, acted as MSs. They were recruited and underwent comprehensive training in role-playing, audio recording, and the clinical aspects of dietary supplements used in OA management. Their proficiency was verified by the research team before data collection. Each pharmacy was visited once between 5:30 PM and 9:00 PM. If a MS was recognized by pharmacy staff, the visit was rescheduled with the alternate MS.

The scenario involved a 23-year-old woman who visited a community pharmacy to request a dietary supplement containing avocado–soybean unsaponifiables (ASU) for knee joint pain. In Step 1, she provided information about the intended user, their medical and medication histories only if the pharmacist explicitly asked. In Step 2, if the pharmacist did not inquire about key details—including the user’s identity, medical and medication histories—the MS stated that the supplement was intended for her 64-year-old father, who has OA, a mechanical heart valve, and is receiving warfarin therapy. She then asked about the safety of using the supplement under these conditions. All conversations were audio-recorded by the MSs, and the pharmacists’ practice was documented.

This scenario was designed to replicate a real-life clinical situation in elderly patients, highlighting an important but often-overlooked aspect of patient care. A clinically significant interaction exists between warfarin and vitamin K-rich components found in soybean and avocado preparations. Warfarin, one of the most commonly prescribed oral anticoagulants, may have its effect diminished when combined with ASU supplements, potentially reducing the International Normalized Ratio (INR) and increasing the risk of thromboembolic events [14–17]. According to RxList.com and Drugs.com (available at https://www.rxlist.com/supplements/avocado.htm#Interactions, https://www.rxlist.com/supplements/soy.htm#Interactions and https://www.drugs.com/food-interactions/warfarin.html), this interaction is classified as moderate. Additionally, package inserts for ASU supplements warn against their concurrent use.

### Assessment criteria

The pharmacists’ performance was evaluated based on established guidelines from the International Pharmaceutical Federation (FIP)/World Health Organization (WHO) for Good Pharmacy Practice [18] and recommendations by the American Society of Health-System Pharmacists (ASHP) regarding dietary supplements [19]. Pharmacists were expected to inquire about the identity of the intended supplement user, obtain medical and medication histories, identify and explain the warfarin–ASU interaction, and recommend safer alternatives.

### Data analysis

All data were analyzed using the Statistical Package for the Social Sciences (SPSS) software, Version 27 (IBM Corp., Armonk, NY, USA). Descriptive statistics were used to summarize all variables. Binary logistic regression was employed to evaluate the association between the dependent (recognition of the warfarin–ASU interaction) and independent (age, gender, years of experience, and pharmacy ownership) variables. A *p*-value of less than 0.05 was considered statistically significant.

## Results

The pharmacists in charge of all 225 active community pharmacies participated in this study. The demographic characteristics of the participating pharmacists are summarized in Table 1.

**Table 1:** Characteristics of the participating community pharmacists

Characteristics	Number of Pharmacists (*N* = 225), *n* (%)
**Gender**	
- Male	118 (52.44%)
- Female	107 (47.56%)
**Age** (Mean ± SD)	33.59 ± 7.91
**Years of experience as a community pharmacist** (Mean ± SD)	9.21 ± 8.00
**Pharmacy owner**	
- Yes	113 (50.22%)
- No	112 (49.78%)

The average counseling duration was 1.00 ± 0.18 min. During 173 visits (76.89%), fewer than two patients were present at the pharmacy (mean ± SD: 0.96 ± 0.96). When the MS requested an ASU supplement, only 15 pharmacists (6.67%) inquired about the intended user. Pharmacists’ overall practice regarding ASU supplement provision are detailed in Table 2.

**Table 2:** Community pharmacists’ practice in providing non-prescription avocado–soybean unsaponifiables supplements

Pharmacy practice	Number of community pharmacists (*N* = 225), *n* (%)	
	Yes	No
**Collecting information**		
Inquiry about the intended user	15 (6.67%)	210 (93.33%)
Obtaining medical history	9 (4.00%)	216 (96.00%)
Obtaining medication history	8 (3.56%)	217 (96.44%)
**Providing information (Step 1)**		
Side effects	0 (0.00%)	225 (100.00%)
Contraindications	0 (0.00%)	225 (100.00%)
Interactions	0 (0.00%)	225 (100.00%)
Identifying warfarin–ASU interaction	7 (3.11%)	218 (96.89%)
Explaining warfarin–ASU interaction	7 (3.11%)	218 (96.89%)
Properly managing warfarin–ASU interaction and recommending avocado/soybean free supplements	3 (1.33%)	222 (98.67%)
Searching for drug information through available resources	0 (0.00%)	225 (100.00%)
Referring to a physician	4 (1.78%)	221 (98.22%)
**Providing information (Step 2: After the mystery shoppers provided the history details) Number of community pharmacists (*N* = 218))**, ***n*** **(%)**		
Identifying warfarin–ASU interaction	29 (13.30%)	189 (86.70%)
Explaining warfarin–ASU interaction	29 (13.30%)	189 (86.70%)
Properly managing warfarin–ASU interaction and recommending avocado/soybean free supplements	22 (10.09%)	196 (89.91%)
Searching for drug information through available resources	2 (0.92%)	216 (99.08%)
Referring to a physician	10 (4.59%)	208 (95.41%)

ASU: Avocado–Soybean Unsaponifiables

In Step 1, only 7 pharmacists successfully identified the potential warfarin–ASU interaction. In Step 2, after receiving additional patient-specific details, the recognition rate increased to 29 pharmacists. However, in both steps, no pharmacists provided further information about additional interactions, adverse effects, or contraindications related to ASU supplements. Only the seven pharmacists who identified the interaction in Step 1 explained how to use the ASU supplement, while the other participating pharmacists provided instructions for use only upon the MS’s request.

Regarding pharmacists’ demographics, in Step 1, no significant associations were found between recognition of the warfarin–ASU interaction and variables including age (odds ratio (OR) = 0.99, 95% confidence interval (CI) = 0.80–1.23, *p* = 0.95), gender (OR = 0.98, 95% CI = 0.18–5.20, *p* = 0.98), years of experience (OR = 1.09, 95% CI = 0.92–1.30, *p* = 0.34), and pharmacy ownership (OR = 0.52, 95% CI = 0.59–4.66, *p* = 0.56). In Step 2, age (OR = 0.86, 95% CI = 0.68–1.08, *p* = 0.20), gender (OR = 0.96, 95% CI = 0.42–2.22, *p* = 0.93), experience (OR = 1.20, 95% CI = 0.95–1.50, *p* = 0.12), and ownership (OR = 0.97, 95% CI = 0.34–2.80, *p* = 0.95) also remained non-significant predictors.

## Discussion

The findings of the present study revealed that pharmacists exhibited a reluctance to engage in patient counseling for dietary supplements, particularly in OA management. Many failed to obtain a comprehensive history, and when the MS provided them with information, their responses were often dismissive—indicating a lack of concern—without verifying details against references or package inserts. They fell short of both professional expectations and international standards. Furthermore, only 15 pharmacists (6.67%) proactively inquired about the intended user of the supplement, indicating deficiencies in clinical training and emphasizing the crucial role of pharmacists in medication safety [20]. The study’s results emphasized the critical importance of thorough patient history-taking, particularly in OTC product counseling. Initially, only 7 out of 225 pharmacists recognized the warfarin–ASU interaction; however, this number increased to 29 in Step 2, following patient history disclosure. This remarkable improvement suggests that proper history-taking can significantly enhance clinical decision-making and medication safety [21].

In the present study, none of the pharmacists provided comprehensive counseling on side effects, contraindications, or drug interactions related to ASU supplements, highlighting a significant gap in patient education [22]. Additionally, only four pharmacists in Step 1 and ten in Step 2 recommended consulting a physician, revealing limited interprofessional collaboration [23]. Another noteworthy concern was the insufficient use of evidence-based resources during pharmacist consultations. Only two pharmacists referred to drug references or package inserts when addressing the ASU-warfarin interaction, emphasizing the need for increased reliance on standardized materials [24]. Notably, manufacturers clearly warn against the concurrent use of ASUs with warfarin, indicating that a straightforward review of package inserts could significantly improve pharmacist awareness.

The previous similar studies on simulated patients regarding OTC medications in Kerman, Iran highlighted the key role of history taking and concluded that pharmacists must strengthen their clinical evaluation and communication skills [12, 13].

A recent study identified heavy workloads, job-related stress, and busy pharmacy environments as barriers to pharmacist–patient interactions [25]. However, these factors do not seem applicable to the current study, given that in 76.89% of the visits, fewer than two customers were present in the pharmacy. Moreover, pharmacists’ unwillingness to provide comprehensive counseling appears unrelated to time constraints. It should be noted that consultation durations in this study averaged under two minutes—well below the recommended minimum of three minutes per patient [26].

A major contributing factor to this suboptimal practice is the lack of financial incentives for pharmacists to provide clinical services. Pharmacists in Iran receive no reimbursement for patient counseling, which discourages active engagement beyond medication dispensing [27]. Internationally, fair and efficient reimbursement models have been recognized as critical for enhancing pharmacy services, ensuring pharmacists integrate patient-centered care into their routine practice [27]. Supporting this perspective, Aggarwal et al. emphasized that extrinsic motivators, such as salary adjustments, professional development opportunities, and performance-based incentives, have been identified as effective drivers of improved healthcare delivery [28].

Community pharmacy faces growing challenges related to public perception. Patients often view pharmacists as medication dispensers rather than healthcare providers, limiting their engagement in clinical decision-making and diminishing their role in patient care [29]. Establishing standardized training programs, continuing education initiatives, and strict adherence to evidence-based guidelines are essential for enhancing pharmacist counseling skills—especially in nutraceutical use. Policymakers should invest in pharmacist education, equipping them with the necessary tools to prevent medication misuse, promote better treatment outcomes, and enhance patient safety. These efforts are crucial for redefining the pharmacist’s role within the healthcare system and strengthening public trust in pharmacy services [22, 30].

### Limitations

This study was conducted in a single city, which may limit the generalizability of the findings. Socioeconomic and cultural differences across regions may influence pharmacy practice. Since the data were collected during evening hours, when pharmacies were less crowded, conducting the study at different times of the day might provide alternative insights into pharmacist-patient interactions.

## Conclusion

Pharmacists provide limited and insufficient counseling on dietary supplements for osteoarthritis, underscoring a significant gap in patient education and the safe use of dietary supplements. Since public health literacy, particularly concerning medications, is closely tied to pharmacists’ expertise, strengthening their clinical training and implementing supportive reimbursement models are crucial for promoting safer, patient-centered pharmaceutical care.

## Data Availability

The datasets used and/or analyzed during the current study are available from the corresponding author upon reasonable request.

## Abbreviations

OA: Osteoarthritis
NSAIDs: Non-steroidal anti-inflammatory drugs
FDA: Food and drug administration
OTC: Over-the-counter
MS: Mystery shopper
ASU: Avocado–soybean unsaponifiables
INR: International normalized ratio
FIP: International pharmaceutical federation
WHO: World health organization
ASHP: American society of health-system pharmacists
SPSS: Statistical package for the social sciences
